# A national COVID-19 quarantine survey and its impact on the Italian sports community: Implications and recommendations

**DOI:** 10.1371/journal.pone.0248345

**Published:** 2021-03-15

**Authors:** Giovanni Fiorilli, Elisa Grazioli, Andrea Buonsenso, Giulia Di Martino, Tsopani Despina, Giuseppe Calcagno, Alessandra di Cagno

**Affiliations:** 1 Department of Medicine and Health Sciences, University of Molise, Campobasso, Italy; 2 Department of Motor, Human and Health Sciences, University of Rome “*Foro Italico*”, Rome, Italy; 3 Faculty of Physical Education and Sports Science, National and Kapodistrian University of Athens, Athens, Greece; Radboud Universiteit Behavioural Science Institute, NETHERLANDS

## Abstract

The outbreak of the 2019 coronavirus disease (COVID-19) created an international public health emergency, challenging the psychological resilience of the general population. Regarding this matter, a web-based survey was performed. Data were collected from the following 1,668 self-selected volunteers: 800 athletes (28.30 ± 10.93 years old); 558 coaches (36.91 ± 11.93 years old); and 310 sports managers (42.07 ± 13.38 years old). To assess the level of psychological stress, an Impact of the Event Scale-Revised (IES-R) questionnaire was used. The results indicated that 34.4% of the participants who were interviewed were affected by subjective distress while 26.4% rated their psychological impact from the sports activity interruption as severe. Separated one-way analysis of variance (ANOVA) tests showed significant differences in the IES-R total score (TS), indicating that the level of stress in terms of gender revealed that women were more stressed than men (p = 0.000), for “sports roles” in which the manager and coaches were more stressed than the athletes (p < 0.05), and “type of sport” in which fitness and individual athletes were more stressed than team athletes (p < 0.01). The middle-level athletes showed significantly more hyperarousal levels than high-level athletes (p = 0.012). The results of this survey may raise awareness of this problem and help athletic associations to have appropriate guidelines in order to better sustain their memberships and organize an optimal resumption of their sports activities. Along these lines, social interactions, which are typical of team sports, are crucial to warrant resilience and psychological health. The athletes by managing independently the new rules and measures, thanks to a clear communication, could improve their adaptive stress reaction.

## Introduction

Athletic endeavors provide several benefits to an individual’s health and well-being and have relevant psychological, cultural, and environmental influences [1]. The outbreak of the coronavirus disease (COVID-19) created an international public health emergency, causing the Italian government to take unprecedented measures to stop the spread of the virus. This included enforcing policies regarding the avoidance of human-to-human transmission via social distancing. Schools as well as recreational, cultural, and sports centers were closed; sporting events and gathering in public spaces were forbidden [2]. This environment led to a thorough modification of sports organizations with unprecedented measures [3].

Sports represent the core activity of an athlete’s weekly routine and their life. Practice is organized daily with frequently scheduled competitions—even throughout the weekends [4]. Home confinement, a result of COVID-19 restrictions, represents a partial or complete level of detraining that will impair an athletic person’s performance and could determine an early conclusion to their career [5]. Evans et al. [6] suggested that this “abrupt stoppage” in this type of activity especially affected older and elite athletes at multiple levels. This included lost revenue or sponsorships and changes to their business strategies along with the disruption of their athletic careers [7]. Notably, this effect is less evident in younger athletes who have a long career ahead of them. They could be more relaxed while waiting for the resumption of sports activities, having time for both expertise development and other sports-related opportunities [8].

The impact of the lockdown has been recognized as a gender dimension [9]. Kogler and colleagues [10] affirmed that males usually showed better ability to cope with stress than women. The athletic woman’s concern from the uncertainty of this period was increased by the observation of gender inequalities with regard to the reduced revenues and the economic priority reserved for the men’s sports necessities [11]. The unpaid work of women increased, especially during this period [12]. Regarding young or amateur athletes, the loss of their usual sports routines could increase the probability of their dropping out, which is caused by the additional cascading of economic effects and financial loss charged to amateur associations and related managers [3]. This unprecedented crisis will involve families who may be in short supply and may have not money or time to provide athletic opportunities for their children once everything gets back to normal [3]. The closure of gyms and fitness centers have forced the modification of exercise programs and the habits of amateur fitness athletes; they have to train at home using minimal gym and/or fitness equipment without supervision by staff or coaches [13].

During the lockdown, coaches had to maintain relationships with their athletes, giving them support with physical conditioning protocols and ensuring frequent contact—valid tools to help them deal with the uncertainty of this period [14]. Even if the Italian government provided an economic incentive to support the coaches during the sports activity suspension, the fear of losing both their incomes and their athletes due to drop-out activity could cause confusion and negative mood swings [15]. Considering the difficulty of predicting when both amateur and elite sports will restart, managers have to be organized during this time and support the coaches and athletes with home-based training. With entrepreneurial thinking, they have to guarantee the sports associations’ “economic survival,” soliciting a progressive implementation of working from home, which allows social distancing. Furthermore, there is the organizing of online fitness events or conferences and encouraging outdoor activities [3]. Finally, there is the damage to the sports industry and its affiliated markets caused by the suspension of tournaments and competitions (upstream and downstream) [16, 17].

Therefore, the psychological impact of the COVID-19 consequences puts the Italian sports community at a psychological health risk. This involves athletes from the youth and amateur levels all the way up to the Olympians and professionals; moreover, it includes the associated staffs, from coaches and physician trainers to managers. This situation is negatively potentiated by the removal of the social support network in relation to the normal training routine suspension. Stressors included longer quarantine duration, financial loss, and stigma [18]. Accordingly, long-lasting effects caused by the quarantine conditions were expected [19–21].

The lockdown consequences could be different in relation to demographic and cultural variables (Italian regions, age, gender, and lifestyle); environmental sports conditions; and their interactions.

A widely used self-report questionnaire for post-traumatic stress was administered in this study to present a formal recognition of stress related to the suspension of sports and the quarantine. The Impact of Event Scale—Revised (IES-R) from Weiss and Marmar [22] is a good tool to differentiate symptom patterns in individuals with low levels of symptoms.

The aim of this web-based survey, which was carried out within the Italian sports community, was to explore the potential influencing factors on the onset of distress from the suspension of sports activity. It would, provide a theoretical basis for psychological and organizational interventions. It was hypothesized that elite athletes could experience high levels of distress due to the fear of their career being disrupted or changes to their sports engagement [23]. These effects were expected to be more dominant for female athletes [12]. However, considering that this sample mainly consists of amateur athletes, different results could be expected.

The concerns of coaches and managers regarding the economic crisis and their athletes’ organization could increase [15]. The economic consequences of the pandemic and the recession from the lowered levels of demand make it difficult to plan for future economic actions [24]. Managers were required to control and reorganize the sports environment—whether or not the activities would resume—using their readiness and capability to cope with the new situation. Managing these processes under considerable uncertainty could lead to maladaptive or positive emotional responses [16].

The results of this survey may raise awareness of this problem and help athletic associations have available and appropriate guidelines for communication and orientation in order to better sustain their memberships and to organize an optimal resumption to their sports activities.

## Methods

### Study design and participants

A cognitive survey was administered during the first phase of the Italian lockdown period (March 12, 2020 to May 3, 2020). The participants were athletes, coaches, and sports managers. The Italian population was recruited through a snowball sampling strategy using an online survey platform (Google Form). The only inclusion criterion was to be affiliate with a national federation and/or a sports association.

The 1,668 self-selected volunteers were as follows: 800 athletes (28.30 ± 10.93 years old); 558 coaches (36.91 ± 11.93 years old); and 310 sports managers (42.07 ± 13.38 years old). Regarding the technical level of the athletes, the low-level ones included amateur athletes who trained themselves twice per week; the middle-level participants included athletes who performed in local competitions and trained themselves more than twice per week; and the high-level athletes performed in national competitions organized by the national federations. The coaches’ sample was divided into three groups based on their professional qualifications, and they were as follows: amateur coaches with no permanent employment status, semi-professional coaches with part-time employment, and professional coaches with full-time jobs and permanent employment status. The managers’ sample was made up of managers who were not professionals. The majority of the athletes did not receive earnings for their sports activities. Regarding the typologies of the sports, it is necessary to consider that generally team-sport athletes have a longer career span than individual ones; for amateur and fitness athletes, sports activities are a part of their active lifestyle.

In the first section, the survey assesses sociodemographic factors such as age; gender; geographical location; “sports role” (athletes, coaches, and managers); “type of sport” (fitness, individual, and team sports); and “technical level” (high, middle, and low) (Table 1).

**Table 1:** Sample characteristics.

Variable	n (%)	
**Total**	**1668 (100.0)**	
**Gender**		
**Athletes**		
Male	426 (53.2)	
Female	374 (46.8)	
**Coaches**		
Male	263 (47.1)	
Female	295 (52.9)	
**Managers**		
Male	189 (61)	
Female	121 (39)	
**Age (mean ± SD)**	33.74 ± 12.97	
**Athletes (mean ± SD)**	28.25 ± 10.97	
Young (≤ 22)	300 (37.5)	
Middle (> 22; < 30)	252 (31.5)	
Adult (≥ 30)	248 (31.0)	
**Coaches (mean ± SD)**	36.90 ± 11.94	
Young (≤ 30)	201 (36.0)	
Middle (> 30; ≤ 45)	206 (37.0)	
Adult (> 45)	151 (27.0)	
**Managers (mean ± SD)**	42.07 ± 13.38	
Young (≤ 35)	113 (36.4)	
Middle (> 35; ≤ 50)	99 (31.9)	
Adult (> 50)	98 (31.6)	
**Role**		
Athletes	800 (48.0)	
Coaches	558 (33.4)	
Managers	310 (18.6)	
**Geographic Area**		
North	375 (22.5)	
Centre	687 (41.2)	
South	606 (36.3)	
**Sports**		
Fitness	236 (14.2)	
Individual	786 (47.1)	
Team	646 (38.7)	
**Technical Level (without managers)**		
**Athletes**		
Low-level	243 (30.4)	
Middle-level	294 (36.7)	
High-level	263 (32.9)	
**Coaches**		
Low-level	183 (32.8)	
Middle-level	185 (33.1)	
High-level	190 (34.1)	

The second section evaluates the level of psychological distress caused by the sports activity suspension, using the IES-R questionnaire [22].

The IES-R questionnaires were widely spread via social networks to the national sports federations, sports organizations, and sports clubs. A cover letter explaining the objective of the study, the assurance of confidentiality and anonymity was added before the questionnaire. The generation of a personal security code warranted the data anonymity; all participants gave their electronic informed consent. The study was designed and conducted in accordance with the Declaration of Helsinki and approved by the bioethical local committee of the University of Rome’s “*Foro Italico”* [University Committee for Research (CAR-IRB), Code: CAR 55/2020]. The psychometric properties of the IES-R Italian version were validated for researches in Italy by Craparo and his colleagues [25].

### Screening questionnaire

The IES-R questionnaire was designed to assess current subjective distress resulting from the withdrawal of sports activities due to the COVID-19 quarantine. It is a self-administered questionnaire composed of 22 items, each with a Likert rating scale from zero to four. The response for each question was 0 (“not at all”), 1 (“rarely”), 2 (“moderately”), 3 (“sometimes”), and 4 (“often”). The total score (TS) range of greater than 32 (cut-off), out of a maximum of 88, identifies the stress subjects. The TS was split into the following: “normal” (score from 0 to 23); “mild” (score from 24 to 32); and “moderate and severe” psychological impact (score > 32) [26]. The questionnaire includes three subscales aimed at measuring the symptoms of intrusion, avoidance, and hyperarousal [27]. Intrusion concerns obsessive and unpleasant images and thoughts related to the traumatic experience. Avoidance concerns the effort of rejecting particular thoughts or feelings from consciousness. This active defensive behavior arises disengagement and insensibility. Hyperarousal symptoms are related to hypervigilance, anger, irritability and low concentration. The subscales scores’ such as intrusion (items 1, 2, 3, 6, 9, 14, 16, and 20), hyperarousal (items 4, 10, 15, 18, 19, and 21) and avoidance (items 5, 7, 8, 11, 12, 13, 17, and 22), indicate the eventual occurrence of these different responses [28].

### Sampling stratification

According to the role covered in the sports association, the participants were divided into athletes, coaches, and managers; according to the sports practiced in team sports, it was individual sports and fitness. Regarding the geographic location of the participants, the sample was divided into three strata (north, center, and south).

### Statistical analysis

Data analysis was performed using SPSS Statistics 21 (IBM) software. The normal distribution of continuous variables was verified using the Kolmogorov-Smirnov test. Data were not normally distributed, so square root transformation was used. Descriptive statistics are presented as mean and standard deviations for continuous variables normally distributed, and percentages are used for categorical variables. Separated one-way analysis of variance (ANOVA) tests were performed to test the differences among the IES-R TS figures as dependent variables. The independent variables were gender; age; sports role (athletes, coaches, managers); geographic area (north, center, and south); type of sport (individual, team, and fitness); and technical level (high, middle, low). A multivariate analysis of variance (MANOVA) test was performed to evaluate the IES-R subscales’ (avoidance, intrusion, and hyperarousal) differences among the groups. Post-hoc comparisons were performed using Fisher’s Least Significant Difference (LSD) test and Bonferroni alpha level correction was applied. Furthermore, 95% of confidence intervals (CIs) for the differences were reported. In addition, the internal consistency of the IES-R was evaluated within the total and subscale scores. The alpha test level for statistical significance for all variables was set at 0.05. Furthermore, Cohen’s d effect size (ES) was calculated for statistically significant differences; values below .49 were considered small effects; values between .5 and .79 were considered medium effects; and values ≥ .8 were considered large effects [29].

## Results

### Total score

The IES-R demonstrated high internal consistency for the total scale (Cronbach’s alpha = 0.92) and an adequate consistency for the three subscales (intrusion = 0.77; avoidance = 0.75; hyperarousal = 0.76).

The whole sample showed the IES-R TS (28.18 ± 14.86), and a total of 574 responders were affected by subjective distress (34.4%). The psychological impact level (PIL) results referring to normal, mild, moderate, and severe ranks are reported in Table 2.

**Table 2:** Total IES-R scores split for psychological impact level- PIL.

PIL	Athletes		Coaches		Managers	
	Total	%	Total	%	Total	%
Normal (0–23)	371	46.4%	248	44.4%	120	38.7%
Mild (24–32)	179	22.4%	102	18.3%	74	23.9%
Moderate & Severe (≥ 33)	250	31.2%	208	37.3%	116	37.4%

Separated one-way ANOVA tests showed significant differences in the IES-R TS for gender, where female athletes and coaches had a higher level of stress than the males (p = 0.000); moreover, no statistical differences were found for gender in the managers. Significant differences were found for sports role where the athletes showed a lower TS compared to both the coaches (mean difference = -2.13; p = 0.025) and the managers (mean difference = -3.04; p = 0.006). No statistical differences were found between the coaches and managers (mean difference = -0.91; p = 1.00). The analysis between the types of sports groups showed a significantly lower TS in team sport athletes than in fitness athletes (mean difference = -4.31; p = 0.000), and between team and individual sports athletes (mean difference = -2.68; p = 0.002). There were no statistical differences between fitness and individual athletes (mean difference = 1.64). No significant differences were found for geographic area, age, and technical level.

### IES-R subscales

Significant differences were found in the IES-R subscales for gender, in which the female athletes showed higher levels than males for all subscales (all p = 0.000). The same results were found for coaches, where females showed significant differences for all subscales (intrusion p = 0.000; avoidance p = 0.001; hyperarousal p = 0.000). Significant differences were found for hyperarousal in managers, with higher scores in females than in males (p = 0.020). Regarding the technical level, the low-level athletes showed higher avoidance scores than the middle-level athletes (mean differences = 1.29; p = 0.016). Instead, the middle-level athletes showed higher scores of hyperarousal than the high-level athletes (mean differences = -1.03; p = 0.012). No statistical differences were found for technical level in the coaches. The analysis for age showed that middle-aged coaches showed higher hyperarousal scores than adults (mean differences = 1.30; p = 0.022). No statistical differences were found for age in the athletes and managers. Despite the significant results, which can be accounted for by the large sample size, the effect size was small.

The MANOVA test showed significant differences between sports role, type of sport, and technical level IES-R subscales. Athletes reported a lower score for intrusion and hyperarousal compared to coaches (intrusion mean differences = -0.94; p = 0.012; hyperarousal mean differences = -0.58; p = 0.045). Comparing athletes and managers, athletes showed a significantly lower score on the intrusion subscale (mean difference = -1.64; p = 0.000). No significant differences were found for the hyperarousal subscale and avoidance subscale among all the groups. Comparing coaches and managers, no significant differences were found in all subscales. Comparing fitness and team, the MANOVA procedure showed significant differences for intrusion (mean differences = 1.42; p = 0.005); avoidance (mean differences = 1.89; p = 0.000); and hyperarousal (mean differences = 1.00; p = 0.007). Comparing team and individual, the MANOVA showed significant differences for intrusion (mean differences = -0.97; p = 0.007); avoidance (mean differences = -0.95; p = 0.003); and hyperarousal (mean differences = -0.76; p = 0.003). No significant differences were found between the fitness and the individual. Even these significant results need to be carefully considered, because the effect size is small. The between-group ANOVA results for the IES-R TS and IES-R subscales are presented in Table 3.

**Table 3:** Comparison among groups for IES-R total score and IES-R subscales.

**IES-R total score**	**Groups**		**Mean ± SD**		**Standard error**	**95% CI**		**p-value**	**ES**
						**Low**	**High**		
	Athletes	Coaches	26.90 ± 14.27	29.03 ± 15.13	0.804	- 4.06	- 0.20	0.025*	0.144
	Athletes	Managers	26.90 ± 14.27	29.94 ± 15.63	0.976	- 5.38	- 0.70	0.006*	0.203
	Coaches	Managers	29.03 ± 15.13	29.94 ± 15.63	1.033	- 3.39	1.57	1.000	
	Fitness	Individual	30.62 ± 16.09	28.99 ± 15.43	1.083	- 4.23	0.96	0.393	
	Fitness	Team	30.62 ± 16.09	26.31 ± 13.44	1.109	0.82	4.54	0.000*	0.290
	Team	Individual	26.31 ± 13.44	28.99 ± 15.43	0.002	- 6.97	- 1.66	0.002*	0.213
**IES-R subscales**	**Groups**		**Mean ± SD**		**Standard error**	**95% CI**		**p-value**	**ES**
						**Low**	**High**		
**Avoidance**	Athletes	Coaches	9.42 ± 5.35	10.02 ± 5.65	0.303	- 1.33	0.12	0.140	
	Athletes	Managers	9.42 ± 5.35	10.21 ± 5.98	0.368	- 1.67	0.09	0.096	
	Coaches	Managers	10.02 ± 5.65	10.21 ± 5.98	0.389	- 1.12	0.75	1.000	
	Fitness	Individual	10.94 ± 5.91	10.01 ± 5.73	0.408	- 0.04	1.91	0.066	
	Fitness	Team	10.94 ± 5.91	9.05 ± 5.17	0.418	0.89	2.89	0.000*	0.340
	Team	Individual	9.05 ± 5.17	10.01 ± 5.73	0.292	- 1.65	- 0.25	0.003*	0.175
**Intrusion**	Athletes	Coaches	10.73 ± 5.90	11.67 ± 6.00	0.328	- 1.73	- 0.16	0.012*	0.158
	Athletes	Managers	10.73 ± 5.90	12.37 ± 6.20	0.398	- 2.60	- 0.69	0.000*	0.295
	Coaches	Managers	11.67 ± 6.00	12.37 ± 6.20	0.422	- 1.71	0.31	0.291	
	Fitness	Individual	12.12 ± 6.39	11.66 ± 6.30	0.442	- 0.60	1.52	0.900	
	Fitness	Team	12.12 ± 6.39	10.70 ± 5.58	0.453	0.34	2.51	0.005*	0.225
	Team	Individual	10.70 ± 5.58	11.66 ± 6.30	0.316	- 1.72	- 0.21	0.007*	0.161
**Hyperarousal**	Athletes	Coaches	6.75 ± 4.25	7.34 ± 4.54	0.238	- 1.15	- 0.01	0.045*	0.134
	Athletes	Managers	6.75 ± 4.25	7.36 ± 4.54	0.289	- 1.30	0.09	0.111	
	Coaches	Managers	7.34 ± 4.54	7.36 ± 4.54	0.306	- 0.76	0.71	1.000	
	Fitness	Individual	7.56 ± 4.95	7.32 ± 4.55	0.321	- 0.53	1.01	1.000	
	Fitness	Team	7.56 ± 4.95	6.56 ± 3.96	0.328	0.22	1.79	0.007*	0.223
	Team	Individual	6.56 ± 3.96	7.32 ± 4.55	0.229	- 1.31	- 0.21	0.003*	0.178

## Discussion

The COVID-19 pandemic and relative quarantine caused the suspension of sports activity at every level. This condition has led to undesirable psychological pressure on the sports community in Italy—likely with long-term consequences [30]. A large number of studies have been published regarding the difficulties and necessities of elite sports contests during the unpredictable and uncontrollable COVID-19 period [5, 9, 31]; however, less or nothing has been taken into consideration regarding amateur sports performers. In Italy, it would be considered that amateur sports involve approximately 14.790,000 athletes and about 1.500,000 coaches and managers [32]. It would be considered that this survey was administered during the first Italian lockdown; however, until now, any intervention was not scheduled for this large part of the population and it still is the case today, even during the second lockdown. All efforts were applied to guarantee health, competition, and training for elite sports, but amateur conditions have been forgotten and set aside for another time. This study aimed to assess the psychological conditions of both elite and amateur performers and provided data to focus attention on the needs of amateur sports in this global phenomenon.

The results indicated that 34.4% of the participants who were interviewed were affected by subjective distress and 26.4% rated their psychological impact from the lockdown as severe. As in previous studies, women appear significantly more vulnerable to distress than men in both the athletes and coaches’ groups [21–33].

Gender differences are evident with regard to both adaptative and reactive behaviors to potential distress for traumatic events [34]. The COVID-19 lockdown influenced the women’s sports environment more than that of the men; athletic women may potentially feel that this situation is more precarious and worrying than men [35]. The impact of reduced investments caused by the economic burdens—particularly in women’s sports—increased the female athletes’ concerns about how their careers could be affected. During the quarantine, female athletes have had difficulties accessing resources such as facilities or health controls that are prioritized for male athletes [35].

Regarding coaches, the financial precariousness of women was exacerbated by the impact of COVID-19. Low salaries, short-term and unstable contracts, and uncertain work conditions are probably expected by female coaches, thus raising their anxiety and distress for their future [7].

This condition is expected also in the general working population in which persistent gender hierarchy, despite major socio-economic transformation, has continued to advantage men over women in status, material resources, and authority. According to previous studies, gender equality could increase economic growth and improve education [36, 37].

Regarding age, significant differences were found between the middle-aged and adult groups, in which younger respondents experienced lower levels of self-reported stress [38]. Considering that age in previous studies represented a protective factor in dealing with stressful situations and that younger subjects were at higher risk of mental health problems [39, 40], our results confirmed the value of physical activity and sports to counteract psychological distress. The middle-aged group of athletes (23 to 30 years old), for example, represented the group that is more worried about their career instability than the other groups, considering that they have not had a long time for their career and probably are expecting to withdraw from their athletic engagement. In addition, the uncertainty about their returning to training gives them a high sense of disappointment as their skills diminish over time [41]. The younger group has a long-term career in front of them, and the loss of a season of training and competition could not represent a problem, whereas the oldest (over 48 years old) could not have great expectations from their sports activities. Consequently, the middle-aged athletes have responded with a negative emotional state. Moreover, considering that the data were collected during the acute phase of the pandemic, the initial emotional response of all the athletic performers—in particular coaches and managers—might be typically from the confusion regarding this unknown, uncertain situation, and developing anxiety [15].

The level of stress of coaches and managers was significantly higher than that of the athletes. In this sample, the coaches and managers mostly organized and supervised amateur clubs, where they remain for a long time, promoting their clubs’ success, of which they are responsible for their survival. Coaches and managers need to be aware of not only the temporary situation due to the lockdown, but also to the social and economic consequences of canceled training and events [17]. Clubs during the COVID-19 pandemic entered into an unspecified period of financial insecurity. Managers will limit the economic impact of earning losses and will sustain the costs of improvements in measures to limit the athletes’ health risks. They must rethink their approach to the athletic organization with regard to whether to pursue elite or recreational sports activities [17]. The psychological distress experienced by coaches and sports managers is comparable to that suffered by all the other categories of workers prevented from working by the COVID-19 pandemic or people who have lost their jobs [42]. The unexpected and abrupt stop due to the COVID-19 pandemic caused an unprecedented shock to the economy. Moreover, the poorer and more vulnerable fraction of the working population seems to be more affected by the lockdown consequences [43]. By analogy with this concept, the amateur sports clubs considered in this study deal with high social costs and a great challenge of survival.

Referring to the type of sport that is practiced, fitness performers showed the highest level of distress. Competitive athletes during their preparation underwent several psychological interventions to increase self-control and manage anxiety in order to enhance their performance. Athletic performance in itself provides a stress buffer effect inducing a sense of competence and self-efficacy that could help in coping with stressors. This “buffering effect” was evident throughout the athlete’s career span [44].

Conversely, there is evidence that fitness performers train themselves to a degree that causes them distress and sometimes they feel unable to stop [45]. Stapleton and her colleagues [46] reported that this kind of athletes develop an “exercise dependence prevalence.” This compulsive trend, characterized by an extreme urge to exercise [47], could lead to major distress from the lack of training; individual sports athletes were more stressed than team-sport athletes. Individual sports athletes are persistent in their training and highly oriented to their competitive outcomes [48]. The training suspension could have generated distress from the stopping of their preparation. Team sports warrant a distribution of roles and responsibilities [49, 50]. The athletes who performed team sports showed more optimism and better skills in managing anxiety and stress control than individual sport athletes [51]. The consequences of home lockdown and of uncertainty for their future activity were perceived as less worrying, being mitigate by the frequent contact with their teammates. This condition represents a recognized protective factor.

As in previous studies, no significant IES-R TS was found in stratified geographical areas [52, 53]. Since the same quarantine measures were put in place throughout Italy, the sample, even if living in geographical areas with different epidemic spread levels, have given similar answers. Considering the low participation of the northern Italy sports community in this survey, the most COVID-19 affected area, it was hypothesized that they were not inclined to participate in this survey.

Athletes showed low values in the subscale scores since they are able to manage levels of intrusive thoughts and shift to the avoidant ones. This behavior allows them to carry out active defensive and protective strategies [54]. Several studies have suggested that exercise participation is inversely correlated with hyperarousal and avoidance symptoms and co-occurring conditions [55, 56]. As previous studies showed, dissociative strategies are common in athletes who tend to separate the problems of their life from those of their performance, as psychological defense. This approach, defined as compartmentalization, could cause the masking of distress in athletes [57, 58]. Moreover, repeated exposure to exercise enhanced stress coping abilities and reduced negative cognitive appraisals [59]. Middle-level athletes showed significantly higher levels of hyperarousal than the high-level athletes (p = 0.012). It has been supposed that athletes could experience a sort of detraining syndrome, resulting in an expected decline in skills, decreased muscle mass, increased body fat, somatic anxiety, and negative mood [60]. High performers are more commonly used to coping with anxiety.

Fitness performers showed higher values in all subscale scores compared with other kinds of athletes. As previously highlighted, fitness performers showed perfectionism and satisfaction from intense effort. They reacted with frequent reminiscence of their activity (intrusion), avoiding (avoidance) of the problem, or with anger and anxiety (hyperarousal) [61]. Conversely, team athletes were less affected by distress [61, 62].

Athletes showed significant lower scores in intrusion, than coaches and managers and in hyperarousal than coaches. It has been demonstrated that they are extremely resilient and tend to reduce painful memories, trying to compartmentalize thoughts regarding the stress event from their daily thoughts [57].

The study offers a cross-section that highlights the effects of the pandemic on certain categories related to sports; however, it is not possible to generalize these results as the significance of the data reported is probably due to the large size of the sample, given that the effect size in most cases was very small.

It is difficult to predict when sports activity will restart in the same conditions which were in place before the COVID-19 pandemic. The practice of sports and other physical activities—even more so in the post COVID-19 period—will continue to play an important role in society, promoting global health and well-being. A new organization of sports activity will be needed, requiring several structural and behavioral changes—mandatory or not—once the emergency ends. Managers, coaches, and athletes would have to meet the important challenge to reclaim their own sports life, even if it is organized in a different scenario, accepting that athletes must train differently or that the financial situation of clubs has changed.

## Conclusions

High psychological distress due to the quarantine restrictions was assessed for the general Italian population [63], in which women or individuals who must avoid their usual activities, generating frustration or boredom [64], were identified as more vulnerable groups. As the results of this study indicated, this condition influenced the Italian sports community at different levels.

Regarding the COVID-19 pandemic, sports practitioners have to consider how this event will modify their lives and careers. To avoid psychological discomfort, it could be advisable to educate athletes, coaches, and sports managers about the range of change and preventive measures. This condition allows to restart in a safe environment once the preventive rules and measures are more permissive.

Social interactions appear crucial to warrant psychological well-being and resilience, in the sports environment as well as in general population. Interactive relationships, serving a buffer function, could support individual resilience during the COVID-19 lockdown [65]. Consequently, clear communication with and involvement of the athletes in managing directives and precautionary measures may facilitate an adaptive stress response [66]. The increase in confidence in the preventive measures may translate to better adherence, thus helping to reduce the fear of the unknown and of the reiteration of the fact as the lockdown [67]. Managers will play a major role in taking part in the organization of the resumption of sports. They have to guarantee the environmental predisposition, compliance with the behavioral rules, the respect of the differences in sports disciplines, and the reduction of huge costs in terms of both actual expenses and the loss of economic opportunities. On the other hand, the coaches will have to encourage and guarantee precautionary and preventive measures for their athletes with shorter training sessions, regular rest periods, and rotating shifts for those who are training. By accepting the new behavioral rules, coaches would be “a point of reference” for their athletes who are involving them in these new behaviors. This condition may mitigate maladaptive coping and depression for lost time and lost athletic opportunities.
